# Drug Delivery from Hyaluronic Acid–BDDE Injectable Hydrogels for Antibacterial and Anti-Inflammatory Applications

**DOI:** 10.3390/gels8040223

**Published:** 2022-04-06

**Authors:** Jon Andrade del Olmo, Leyre Pérez-Álvarez, Virginia Sáez Martínez, Sandra Benito Cid, Raúl Pérez González, José Luis Vilas-Vilela, José María Alonso

**Affiliations:** 1 i+Med S. Coop. Parque Tecnológico de Álava, Albert Einstein 15, Nave 15, 01510 Vitoria-Gasteiz, Spain; jandrade@imasmed.com (J.A.d.O.); vsaez@imasmed.com (V.S.M.); sbenito@imasmed.com (S.B.C.); rperez@imasmed.com (R.P.G.); 2 Grupo de Química Macromolecular (LABQUIMAC), Departamento de Química Física, Facultad de Ciencia y Tecnología, Universidad del País Vasco UPV/EHU, 48940 Leioa, Spain; leyre.perez@ehu.eus (L.P.-Á.); joseluis.vilas@ehu.eus (J.L.V.-V.); 3 BCMaterials, Basque Center for Materials, Applications and Nanostructures, UPV/EHU Science Park, 48940 Leioa, Spain

**Keywords:** hyaluronic acid, injectable hydrogels, biocompatibility, drug delivery, antibacterial, anti-inflammatory

## Abstract

Hyaluronic acid (HA) injectable biomaterials are currently applied in numerous biomedical areas, beyond their use as dermal fillers. However, bacterial infections and painful inflammations are associated with healthcare complications that can appear after injection, restricting their applicability. Fortunately, HA injectable hydrogels can also serve as drug delivery platforms for the controlled release of bioactive agents with a critical role in the control of certain diseases. Accordingly, herein, HA hydrogels were crosslinked with 1 4-butanediol diglycidyl ether (BDDE) loaded with cefuroxime (CFX), tetracycline (TCN), and amoxicillin (AMX) antibiotics and acetylsalicylic acid (ASA) anti-inflammatory agent in order to promote antibacterial and anti-inflammatory responses. The hydrogels were thoroughly characterized and a clear correlation between the crosslinking grade and the hydrogels’ physicochemical properties was found after rheology, scanning electron microscopy (SEM), thermogravimetry (TGA), and differential scanning calorimetry (DSC) analyses. The biological safety of the hydrogels, expected due to the lack of BDDE residues observed in ^1^H-NMR spectroscopy, was also corroborated by an exhaustive biocompatibility test. As expected, the in vitro antibacterial and anti-inflammatory activity of the drug-loaded HA-BDDE hydrogels was confirmed against *Staphylococcus aureus* by significantly decreasing the pro-inflammatory cytokine levels.

## 1. Introduction

Hyaluronic acid (HA) glycosaminoglycan composed of β-1,4-glucoronic acid and β-1,3-N-acetyl-D-glucosamine units is a natural, biodegradable, and biocompatible polysaccharide present in biological fluids and tissues [1]. These advantageous properties have turned HA into an industrially relevant biopolymer in the biomedical sector [2]. Over the last few years, although HA commercialization has focused on the cosmetic/aesthetic field (e.g., dermal fillers, creams, and beauty masks) [3], its applicability has expanded to novel biomedical areas, such as regenerative medicine [4], cancer therapy [5], ophthalmology [6], traumatology [7], and implantology [8,9].

This commercial diversification has occurred due to the development of HA-based smart polymeric networks, taking advantage of the diverse reactive functional groups present in the structure of this polymer [10]. One of these systems is chemically crosslinked HA injectable hydrogels, which, apart from preserving the beneficial properties of HA, show an improved half-life after injection into the human body, with longer residence times than HA solutions [11]. The highly hydrophilic, elastic, and deformable tridimensional polymeric network offers a unique tremendous swelling capacity that enables the absorption and entrapment of large amounts of water with dissolved active agents for subsequent release into specific therapeutic targets [12]. Indeed, sustained drug delivery has become a promising approach to improve the performance of passive biomaterials administrating, in a space–time-controlled manner, encapsulated bioactive agents [13,14], including drugs, proteins, nucleic acids, growth factors, cells, and genes.

The chemical crosslinking of HA can be achieved by numerous crosslinking agents [15]; however, undoubtedly, 1,4-butanediol diglycidyl ether (BDDE) is the most suitable [16]. In fact, the optimal rheological properties along with the adequate injectability and stability of HA-BDDE hydrogels have led to the manufacture of more than 20 and 30 commercial products in the United States and Europe, respectively [17].

However, although HA-BDDE hydrogels are considered to be safe, it has been widely reported that the mere injection of hydrogels into the human body can unexpectedly induce adverse reactions due to differences in their intrinsic properties, such as impurities or the poor quality of the crude material [18]. More frequently, local adverse events after injection are caused by the malpractice of medical doctors or by the injection technique itself (e.g., poor quality operations or postoperative nursing, inappropriate use of needles, rapid injection, rapid flow rates, extremely high injection volumes, multiple punctures, or deep subcutaneous injections) [19]. This usually concludes in chronic pain, bacterial infections, and aseptic inflammations, putting the patient’s health at risk [20,21,22]. These diseases can usually be successfully treated with antibiotics, such as cefuroxime (CFX) [23], tetracycline (TCN) [24], and amoxicillin (AMX) [25], with a demonstrated antibacterial efficacy against Gram-positive and Gram-negative bacteria. Likewise, acetylsalicylic acid (ASA) [26,27] has been demonstrated to be an ideal anti-inflammatory agent for regulating uncontrolled inflammation for many decades.

In this sense, HA-BDDE injectable hydrogels can be loaded with antibiotics and non-steroidal anti-inflammatory drugs (NSAIDs) and, in this manner, be used to effectively treat harmful bacterial infections [28,29] and immune-mediated painful inflammatory responses [30]. Taking into account all of the above, different HA-BDDE formulations were synthesized to obtain long-term biocompatible gels with tunable physicochemical properties. Moreover, the CFX, TCN, AMX, and ASA release profiles from HA-BDDE samples were studied. Finally, drug-loaded hydrogels were tested to evaluate their antibacterial performance against *S. aureus* and their anti-inflammatory activity, quantifying pro-inflammatory cytokines. Hence, it is hypothesized that drug-loaded HA-BDDE formulations may possess a promising applicability in the biomedical industry by reducing harmful bacterial infections and excessively painful inflammations that appear after the injection of hydrogels into the human body.

## 2. Results and Discussion

### 2.1. Synthesis of HA-BDDE Hydrogels

**Figure 1:**
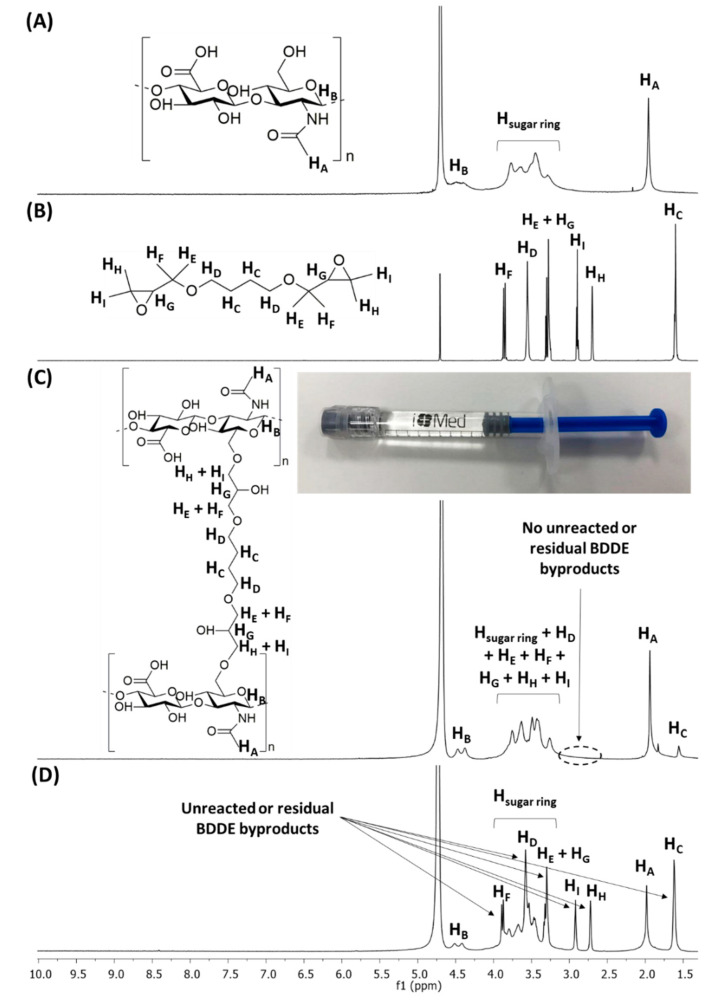
^1^H-NMR spectra of (**A**) HA and (**B**) BDDE and HA-BDDE gels (**C**) after and (**D**) before the dialysis process. Solvent: D_2_O (4.70 ppm).

HA-BDDE hydrogels were obtained after a ring-opening etherification by the nucleophilic attack of HA reactive hydroxyl groups in basic medium on the bisepoxide electrophilic functionalities of BDDE, resulting in the formation of 1,4-butanediol di-(propan-2,3-diolyl) ether bonds [31]. ^1^H-NMR spectroscopy was utilized to verify the crosslinking of HA-BDDE hydrogels (Figure 1) and quantify their degree of modification (MoD) (Table 1, Figures S1–S6).

**Figure 2:**
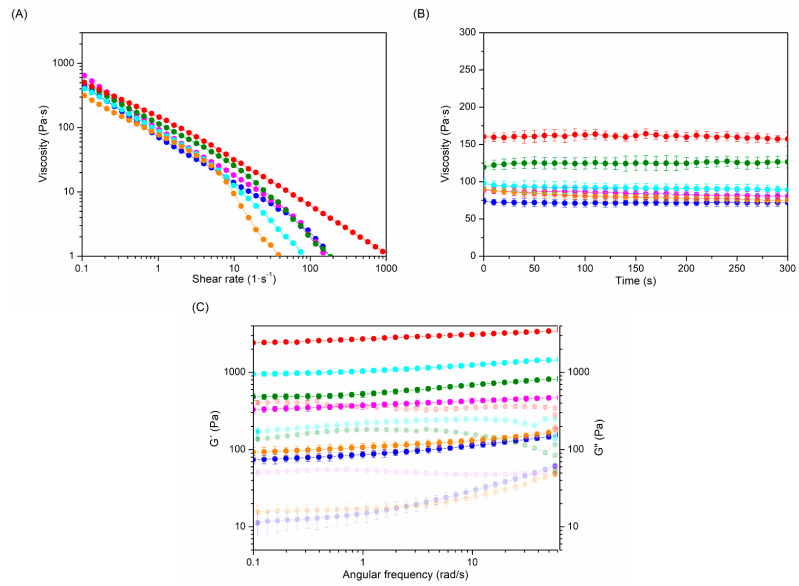
(**A**) Shear rate, (**B**) time, and (**C**) frequency sweeps for viscosity, elastic (G′) and viscous (G′′) moduli determination of HA-BDDE samples. HA-BDDE-1 (●), HA-BDDE-2 (●), HA-BDDE-3 (●), HA-BDDE-4 (●), HA-BDDE-5 (●) and HA-BDDE-6 (●).

Figure 1 shows the ^1^H-NMR spectra of HA, BDDE, and synthesized HA-BDDE hydrogels. The ^1^H-NMR signals of HA (Figure 1A) are displayed at 1.90 ppm (H_A_), 4.50 ppm (H_B_), and 3.20–4.00 ppm (H_sugar ring_), whereas the ^1^H-NMR signals of BDDE (Figure 1B) appeared at 1.60 ppm (H_C_), 2.70 ppm (H_H_), 2.85 ppm (H_I_), 3.30 ppm (H_E_ + H_G_), 3.60 ppm (H_D_), and 3.90 ppm (H_F_). The appearance of a signal corresponding to BDDE alkyl protons (H_C_) after crosslinking at 1.60 ppm (Figure 2C) as well as the non-existence of unreacted BDDE at 2.70–2.85 ppm (Figure 2D) demonstrates the success of the HA-BDDE hydrogel synthesis and the effective removal of residual BDDE. Peak assignments were endorsed by prior NMR analyses [32].

H_C_ protons (1.60 ppm) and methyl protons of the N-acetyl groups (1.90 ppm) were integrated to measure MoD (Table 1, Figures S1–S6). As can be observed in Table 1, the reaction time and BDDE concentration had significant effects on the MoD values. While the HA-BDDE-1 formulation (2 h, 0.5 C) exhibited a lower MoD (13.50%), the increase in the reaction time to 3 h slightly augmented the MoD (HA-BDDE-4, 16.50%). Similarly, the increase in the quantity of BDDE to 2 C (HA-BDDE-3) produced an augmentation of the MoD up to 65.25%. According to the reaction conditions employed, the highest MoD value (76.50%) corresponded to HA-BDDE-6 hydrogel (3 h, 2 C)

Moreover, FTIR was also employed to corroborate the hydrogel synthesis by analyzing the most noticeable vibration bands of HA, BDDE, and HA-BDDE (Figure S7). The increases in the intensities of the peaks seen at 1050–1150 cm^−1^ (C–O–C + C–O stretch) and 2800–2950 cm^−1^ (C–H stretch) verify the establishment of stable ether bonds after the crosslinking reaction. The disappearance of the peaks corresponding to BDDE at 2800–2900 cm^−1^ (C–H stretch) and 800–900 cm^–1^ (C–O stretch) also proves the reaction between the BDDE epoxy ring and HA hydroxyl groups, as well as the effective elimination of unreacted BDDE. Moreover, the consumption of HA intermolecular –OH groups in the HA-BDDE hydrogel led to a decrease in peak intensity at 3400 cm^−1^, whereas at 3050–3300 cm^−1^ the width of the peak increased due to the formation of intramolecular –OH groups and H bonds.

### 2.2. Rheological Properties

Rheological behavior is considered the most important property of injectable hydrogels, since it seriously affects their biomedical applicability [33]. The rheological performance of hydrogels can be modulated by varying their crosslinking degree. Consequently, the effect of the crosslinking degree on the rheological properties of the gels was investigated (Figure 2, Table 1).

**Table 1:** MoD and viscoelastic features of the produced HA-BDDE hydrogels.

Hydrogels	Reaction Time (h)	BDDE Concentration	MoD (%)	Viscosity (Pa·s)	G′ (Pa)	G′′ (Pa)	tan δ	G*
HA-BDDE-1 (●)	2	0.5 C	13.50	71.9 ± 5.0	103.9 ± 4.7	27.1 ± 2.5	0.261	107.4
HA-BDDE-2 (●)	2	C	31.50	84.3 ± 5.7	385.7 ± 14.9	71.6 ± 3.6	0.186	392.3
HA-BDDE-3 (●)	2	2 C	65.25	91.7 ± 6.1	1142.0 ± 58.4	204.4 ± 9.3	0.179	1160.1
HA-BDDE-4 (●)	3	0.5 C	16.50	80.2 ± 5.1	123.2 ± 5.5	24.7 ± 2.8	0.201	125.6
HA-BDDE-5 (●)	3	C	36.75	124.8 ± 8.2	615.8 ± 20.2	107.7 ± 7.5	0.174	625.1
HA-BDDE-6 (●)	3	2 C	76.50	160.9 ± 10.6	2911.1 ± 84.1	354.6 ± 22.1	0.122	2932.5

In all hydrogels, viscosity decreases as the shear rate increases, revealing a shear thinning behavior that confirms the injectability of hydrogels (Figure 2A) [34,35]. Moreover, an elastic modules (G′) higher than the viscous modules (G′′) at all tested frequencies, tan δ values near to 0, and a G′ close to the complex modules (G*) prove that the elastic behavior was the main factor affecting the rheological performance (Figure 2B,C and Table 1) of synthesized gel-like samples.

The rheological parameters of HA-BDDE hydrogels depend on the synthesis conditions. In this regard, increasing the reaction time (from 2 h to 3 h) and BDDE amount (from 0.5 C to C or 2 C) produces a higher viscosity, G′, G′′, and G* and a lower tan δ in the hydrogels as the MoD increases. In fact, when the reaction time was increased from 2 h (HA-BDDE-1, 13.50% MoD) to 3 h, (HA-BDDE-4, 16.50% MoD) the rheological performance improved, with viscosity, G′, and G′′ values of 80.2 ± 5.1 Pa·s, 123.2 ± 5.5 Pa, and 24.7 ± 2.8 Pa, respectively. HA-BDDE-6 exhibited a higher increase in rheological parameters (76.50% MoD, viscosity = 160.9 ± 10.6 Pa·s, G′ = 2911.1 ± 84.1 Pa, and G′′ = 354.6 ± 22.1 Pa) due to the use of the largest amount of BDDE. Overall, these rheological results are in accordance with those achieved for other HA injectable commercial products that have been developed over the last few years in the biomedical industry [36,37].

### 2.3. Morphology, In Vitro Swelling and Degradation, and Thermal Characterization

**Figure 3:**
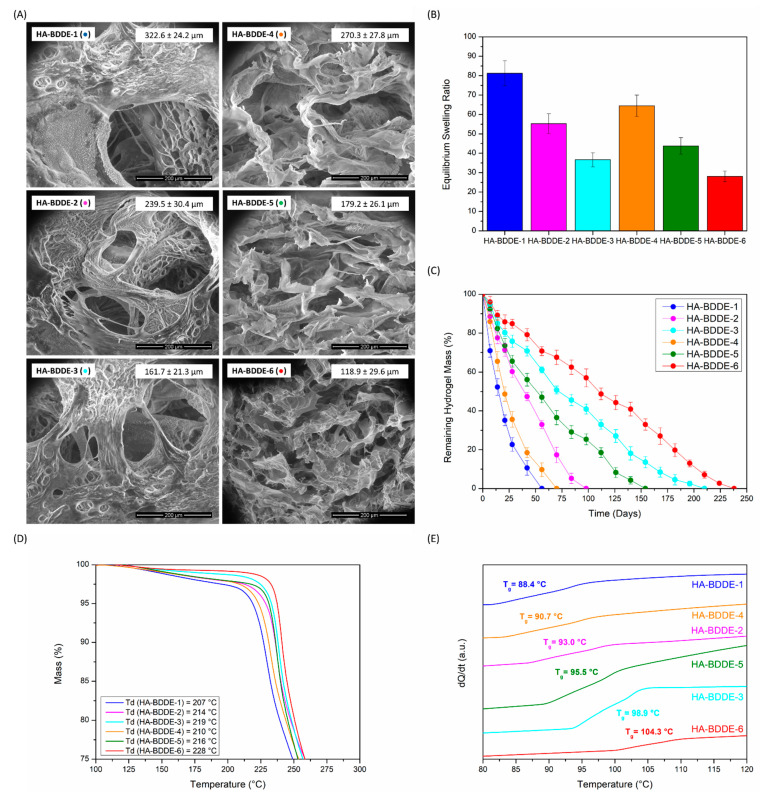
(**A**) LVSEM images and pore sizes, (**B**) swelling ratios, (**C**) degradation profiles, (**D**) TGA curves, and (**E**) DSC thermograms of synthesized gels. HA-BDDE-1 (●), HA-BDDE-2 (●), HA-BDDE-3 (●), HA-BDDE-4 (●), HA-BDDE-5 (●) and HA-BDDE-6 (●).

Morphological observations were performed on freeze-dried HA-BDDE hydrogels (Figure 3A) using LVSEM. Differences in pore sizes directly related to the crosslinking density of the hydrogels could be observed. Indeed, hydrogels synthesized for 3 h (HA-BDDE-4, HA-BDDE-5, and HA-BDDE-6) with a higher MoD display smaller pore sizes than those reacted for 2 h (HA-BDDE-1, HA-BDDE-2, and HA-BDDE-3). Moreover, for both of the reaction times, when 0.5 C BDDE (MoD ↓) was used, the largest pore sizes were measured (322.6 ± 24.2 µm for HA-BDDE-1 and 270.3 ± 27.8 µm for HA-BDDE-4). Afterwards, a significant pore size decrease of about ~90 µm occurred for C BDDE (HA-BDDE-2, HA-BDDE-5). Correspondingly, the use of HA-BDDE-3 and HA-BDDE-6 hydrogels synthesized with the quantity of 2 C BDDE led to the lowest pore sizes (161.7 ± 21.3 µm and 118.9 ± 29.6 µm). In any case, these polymeric microstructures enable the entrapment of large amounts of water for potential drug delivery applications. Hence, the equilibrium swelling ratio and hydrolytic degradation profiles, which play a crucial role in the sustained release of bioactive agents, were measured.

All hydrogels demonstrated highly remarkable swelling (Figure 3B) owing to the various hydrophilic groups present in the chemical structure of HA, which lead to distinctive water absorption and hydration capabilities. HA-BDDE-1 and HA-BDDE-4 samples with a low degree of crosslinking and larger pore sizes are clear examples of this, with equilibrium swelling ratio values of 81.25 ± 6.53 and 64.49 ± 5.50 being reached. Even highly crosslinked HA-BDDE-3 and HA-BDDE-6 hydrogels with smaller pore sizes, which offer a lower swelling ability as a result of the increase in MoD, possess satisfactory equilibrium swelling ratio values (36.68 ± 3.66 and 28.05 ± 2.71).

Regarding the degradation profiles seen in Figure 3C, a direct relation between hydrogels’ stability and their crosslinking density was noticed. At both reaction times, the stability of the hydrogels was progressively increased as the employed BDDE concentration increased from 0.5 C (HA-BDDE-1 and HA-BDDE-4) to C (HA-BDDE-2 and HA-BDDE-5) and 2 C (HA-BDDE-3 and HA-BDDE-6). Increasing the reaction time from 2 h to 3 h also led to a lower degradation rate owing to the higher MoD (HA-BDDE-4 > HA-BDDE-1, HA-BDDE-5 > HA-BDDE-2, and HA-BDDE-6 > HA-BDDE-3). Thereby, hydrogels with the lowest MoD (0.5 C) were completely degraded after 56 days (HA-BDDE-1) and 70 days (HA-BDDE-4), while hydrogels with the highest MoD (2 C) were not completely deteriorated until 210 days (HA-BDDE-3) and 238 days (HA-BDDE-6). Hydrogels synthesized with a C BDDE concentration (30–40% MoD) displayed moderate degradation rates and did not degrade completely until 98 (HA-BDDE-2) and 154 (HA-BDDE-5) days.

Lastly, the thermal degradation (T_d_) and glass transition (T_g_) temperatures were measured using thermogravimetry (TGA) and differential scanning calorimetry (DSC) to assess their influence on the microstructure of polymeric networks. As a matter of fact, a greater degree of crosslinking (↑ MoD) diminishes the ability to move polymeric chains; hence, more energy is required both for these substances to be thermally degraded and for them to move from glassy to viscous conditions (↑ T_d_ and T_g_ values). As observed in Figure 3D,E, a single step of thermal degradation (T_d_) can be viewed at 200–220 °C in thermogravimetric curves, and the T_g_ values ranged from 88.4 to 104.3 °C. This way, the T_d_ and T_g_ values obtained corroborated the existence of this tendency in the physicochemical results.

A clear correlation between the synthesis parameters that influence the crosslinking degree of the gels and their morphology, swelling, and hydrolytic and thermal stability was found, indicating the feasibility of tailoring these key properties according to the desired application of the biomaterial. Hydrogels synthesized for 3 h (HA-BDDE-4, HA-BDDE-5, and HA-BDDE-6) possess enhanced rheological and stability properties as well as extraordinary swelling abilities. Thus, biocompatibility, drug delivery, antibacterial, and anti-inflammatory assays were carried out on these samples.

### 2.4. Biocompatibility Test

**Figure 4:**
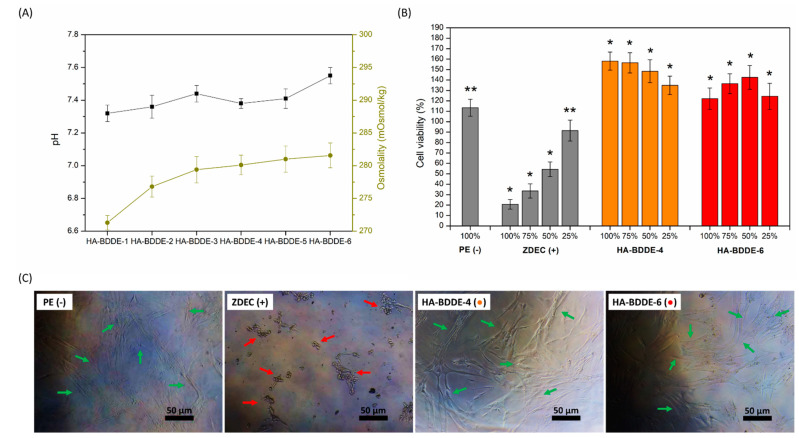
(**A**) Osmolality and pH values of HA-BDDE gels, (**B**) CCL-171 cell viabilities, and (**C**) photographs of damaged (↑, rounded) and healthy (↑, elongated) cells at 100% concentration of PE (−), ZDEC (+), HA-BDDE-4 (●), and HA-BDDE-6 (●). * Significant differences (*p* < 0.05), ** non-significant differences (*p* > 0.05).

It is well known that HA-BDDE injectable hydrogels require a final dialysis purification step to remove harmful unreacted BDDE or by-products [38,39]. In fact, the Food and Drug Administration has an extremely strict tolerable limit specification for commercialized injectable hydrogels (residual BDDE < 2 ppm) [40]. Thus, with the aim of confirming the efficient purification of synthesized hydrogels and their subsequent biological safety, a biocompatibility test was carried out according to the quality standards and limits established by the European Pharmacopeia (Ph. Eur.) and UNE-EN-ISO international standards (Figure 4).

The osmolality and pH of the HA-BDDE formulations were assessed according to the tolerable ranges set by the Ph. Eur. and UNE-EN ISO criteria. In surgical medical devices, a pH range of 6.8–7.6 (7.2 ± 0.4) and osmolality values of 200–400 mOsmol·kg^–1^ are well accepted [41], while in intra-articular formulations osmolality values close to those of blood plasma (285–480 mOsmol·kg^–1^) are tolerated [42]. Therefore, it can be concluded that the pH and osmolality values of HA-BDDE hydrogels are suitable (Figure 4A), as they are within the previously mentioned critical limits. As expected, slightly crosslinked hydrogels (HA-BDDE-1) showed lower values (7.32 ± 0.05, 271.3 ± 1.1 mOsmol·kg^–1^), while highly crosslinked systems (HA-BDDE-6) exhibited higher ones (7.55 ± 0.04, 281.6 ± 1.9 mOsmol·kg^–1^).

In addition, an in vitro cytotoxicity test was carried out via the extraction method in accordance with the UNE-EN ISO 10993-5:2009 standards [43]. These standards specify that cell viabilities greater than 70% indicate the non-cytotoxic behavior of tested biomaterials. HA-BDDE-4 (0.5 C) and HA-BDDE-6 (2 C) were selected to undergo cytotoxicity testing, as it is well known that the quantity of crosslinking agent used is an important risk parameter to consider when it comes to ensuring the biocompatibility of chemically crosslinked injectable hydrogels [44]. Thus, the cytocompatibility of HA-BDDE-4 and HA-BDDE-6 samples was demonstrated by their 158.12 ± 8.65% and 122.10 ± 10.19% cell viabilities (Figure 4B). These significant (*p* < 0.05) cell viability increases not only prove the cytocompatibility of hydrogels but also demonstrate that they promote cell growth and proliferation. The morphology assessment of cells also validated the hydrogel cytocompatibility results with the presence of healthy (↑, elongated) cells (Figure 4C).

Finally, an in vivo acute systemic toxicity test was performed as established in the UNE-EN-ISO 10993-11:2017 standard [45] to evaluate the response of mice to a systemic injection of HA-BDDE hydrogel (Table S1). After injection, all mice manifested a normal weight increase with no considerable systemic toxicity or behavioral or clinical symptoms. Even at macroscopic scale, neither lesions, anomalous changes, nor dead animals were detected. Thus, the HA-BDDE-4 sample satisfies the requirements of the UNE-EN-ISO 10993-11:2017 standard.

All in all, the biocompatibility results demonstrate HA-BDDE hydrogels to be safe injectable biomaterials. Indeed, apart from not observing unreacted BDDE or by-products in H-RMN spectra, the hydrogels fully satisfied the biocompatibility safety requirements and specifications set by the Ph. Eur. and UNE-EN-ISO international norms.

### 2.5. Drug Loading and Release Studies

**Figure 5:**
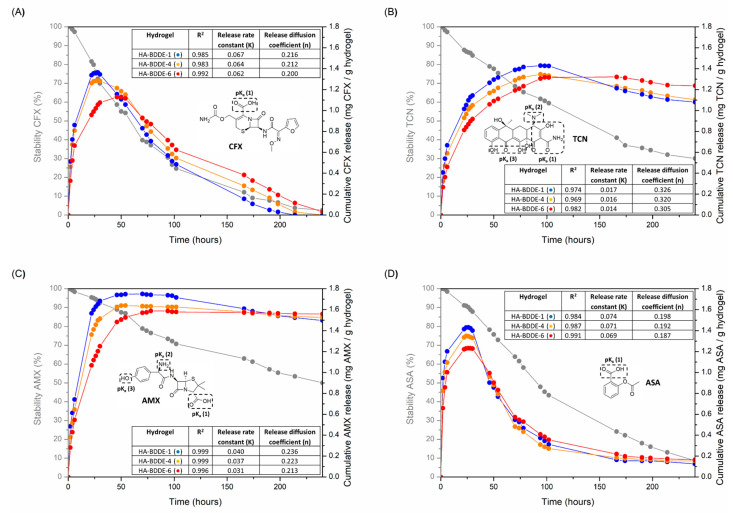
Release of (**A**) CFX, (**B**) TCN, (**C**) AMX, and (**D**) ASA from HA-BDDE-1 (●), HA-BDDE-4 (●), and HA-BDDE-6 (●) gels Release rate constant (K) and release diffusion coefficient (n) values measured by the Korsmeyer–Peppas model are also presented.

Sustained drug delivery is a powerful approach with which to endow hydrogels with advantageous antibacterial and anti-inflammatory activities and, thus, combat adverse effects after injection into the human body [46,47]. Accordingly, CFX, TCN, and AMX antibiotics and the ASA anti-inflammatory agent were separately loaded and released in physiological conditions (PBS, 37 °C) from different hydrogel formulations (Figure 5) in order to analyze the effect of reaction time (HA-BDDE-1 vs. HA-BDDE-4) and BDDE quantity (HA-BDDE-4 vs. HA-BDDE-6) on their drug delivery properties.

**Figure 6:**
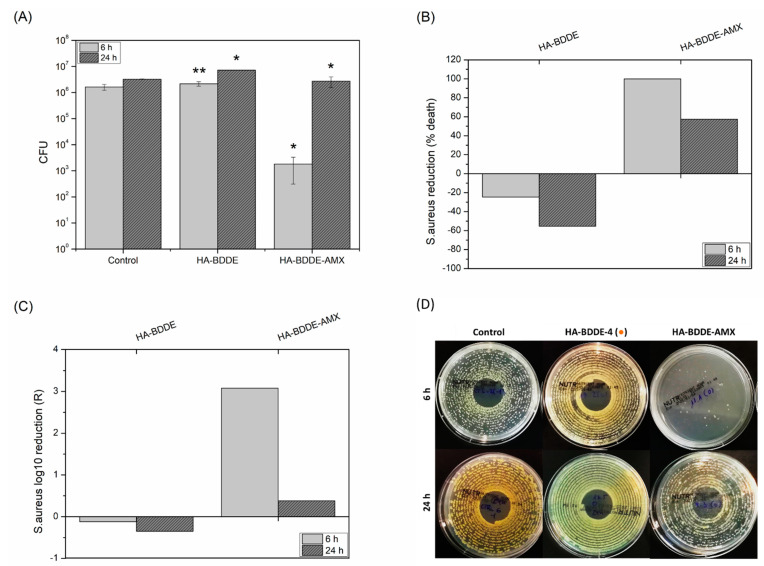
(**A**) Colony Forming Units, (**B**) bacterial reduction (% death), and (**C**) log10 reduction (R) for control, HA-BDDE-4, and HA-BDDE-AMX. (**D**) Images of *S. aureus* agar plates. * Significant differences (*p <* 0.05), ** non-significant differences (*p >* 0.05).

Generally, medical diseases derived from bacterial infections and excessive inflammatory responses are known to occur during the early period of biomedical interventions (<24 h) [48,49]. After that, short- and medium-term drug reservoir action is still necessary and can be achieved by the sustained release of specific low to high doses of drugs. The fact that the long-term release of too-high doses of drugs can lead to serious health problems (e.g., the appearance of drug-resistant bacteria or drug overdose toxicity) must be taken into account [50]. Pharmacologic half-life (t_1/2_) values of 62 h, 135 h, 240 h, and 88 h make CFX, TCN, AMX, and ASA (Figure 6A–D) ideal candidates to ensure spontaneous degradation after pharmacological action.

At pH 7.4, the physicochemical properties of each drug (Table S2) and HA-BDDE hydrogels also have significant effects on the obtained release profiles. Indeed, CFX and ASA are completely negatively charged (− −), TCN is positively ionized (+), and AMX is also negatively ionized but to a lesser degree (−). Additionally, pKa values of ~3–4 for the carboxylic acid moieties present in each glucuronic unit of HA lead to the negative ionization of HA-BDDE polymeric networks at neutral pHs [51].

Considering their drug release profiles, CFX (Figure 5A) and ASA (Figure 5D) displayed similar release profiles because of their similar negative net charge (− −) and t_1/2_. In this regard, the electrostatic repulsion between HA-BDDE hydrogels and both drugs favor a sudden and fast release with a maximum dosage at 24 h (1.05–1.38 mg of CFX and 1.22–1.43 mg of AAS per hydrogel gram). At this time, the greater amount of ASA released is a consequence of its lower molecular weight compared to CFX. Even so, the effect of the different molecular weights can be better appreciated at short test times (6 h), in which 1.00–1.21 mg of ASA per gram of hydrogel is released while only 0.66–0.85 mg of CFX per gram of hydrogel is delivered. Lastly, their low t_1/2_ values lead to their almost total degradation after 175 h.

In contrast, the higher stability (t_1/2_↑) and more positive net charges of TCN (Figure 5B) and AMX (Figure 5C) promote prolonged drug releases with maximum drug doses at longer periods. In particular, negatively charged HA-BDDE hydrogels contribute to retaining more positively charged drugs. In such a manner, as the positive charges of drugs increased (TCN > AMX) a more sustained release was obtained with prolonged therapeutic ranges. Hence, maximum dosages of 1.56–1.74 mg AMX and 1.31–1.42 mg TCN per gram of hydrogel were achieved at 48 h and 100 h, respectively.

Taking into account the aforementioned quantities of drugs released from the HA-BDDE samples, how long each drug takes to reach a concentration that inhibits bacteria colonies is debatable. This characteristic parameter of antibiotics is called minimum inhibitory concentration (MIC) and, according to several authors, 1.5, 0.1, and 0.2 mg·L^−1^ are the MIC values against *S. aureus* for CFX, TCN, and AMX, respectively [52,53]. Therefore, it can be observed that all antibiotics are able to reach their MIC from practically 2 h and maintain it for more than 10 days (Figure 5A–C). On the contrary, although ASA NSAID is mainly used for anti-inflammatory purposes, it can also inhibit *S. aureus* bacteria in spite of possessing a higher MIC (2.5–5.0 mg·mL^−1^) [54].

Moreover, the effects of crosslinking reaction time (HA-BDDE-1 vs. HA-BDDE-4) and BDDE feed (HA-BDDE-4 vs. HA-BDDE-6) on the cumulative release profiles of drugs were also evaluated. Specifically, the sample that was previously demonstrated to have a lower MoD and larger pore size (HA-BDDE-1) showed a faster drug release and higher maximum drug dose. Accordingly, as the MoD increased and the pore size decreased, the ability to release drugs became more restricted, with the HA-BDDE-6 sample being the formulation with the lowest drug delivery capability.

The Korsmeyer–Peppas semi-empirical model [55,56] was employed to determine the main phenomenon that caused the release of drugs from HA-BDDE hydrogels. Accordingly, the overall results obtained from the processed release profiles (Figure 5) for the Korsmeyer–Peppas model supported Fickian diffusion (Case I, n < 0.45) as the predominant release mechanism of drugs. Thus, HA-BBDE injectable hydrogels have been confirmed as adequate platforms for the controlled release of a wide variety of drugs with different therapeutical ranges and maximum doses that can provide antibacterial and anti-inflammatory properties.

### 2.6. Antibacterial Activity

Bacterial infections are a likely cause of adverse reactions to injectable hydrogels; therefore, it is imperative for HA-BDDE biomaterials to have antibacterial activity for successful injection [57,58]. Although the high hydrophilicity and negative charge of HA promote potential antiadhesive and bacterial repelling properties (e.g., HA antifouling coatings on biomedical implants), [59] it is well known that HA-based medical devices are not able to kill bacteria on their own. For this reason, after validating hydrogels as appropriate antibiotic release systems, an antibacterial test was carried out to investigate their biocidal properties against *S. aureus*, one of the most common pathogen that produces clinical infections [60]. The most critical periods of time (6 h and 24 h) for bacterial adhesion and proliferation bioprocesses were chosen for performing an antibacterial test [61,62]. Moreover, for simplicity, only AMX-loaded hydrogel (HA-BDDE-AMX) was selected for use in this study (Figure 6), because, despite novel antibacterials having been developed over the last few decades, AMX is still one of the most extensively prescribed antibiotics [63].

Figure 6 shows that, as expected, HA-BDDE hydrogel was not able to kill bacteria. In fact, the Colony Forming Units (CFU) were increased (2.17 × 106 ± 0.42 × 106 CFU for 6 h and 7.26 × 06 ± 0.04 × 106 CFU for 24 h), causing bacterial population rises of 24.64% and 55.40% for 6 h and 24 h, respectively. This phenomenon is in agreement with previous studies, in which it was concluded that bacteria similar to *S. aureus* tend to colonize hydrogel biomaterials as a nidus for infection after injection [64]. Nevertheless, the HA-BDDE-AMX formulation provoked significant (*p* < 0.05) reductions in viable bacteria compared to the HA-BDDE sample: 99.92% at 6 h (1.82 × 103 ± 1.50 × 102 CFU after releasing 0.65 mg AMX per gram of hydrogel) and 57.38% at 24 h (2.73 × 106 ± 1.20 × 106 CFU after releasing 1.44 mg AMX per gram of hydrogel). This abnormal antibacterial activity decreased as AMX was released and hydrolytically degraded can be ascribed to beta-lactam penicillinases. In this way, *S. aureus* becomes more resistant as beta-lactam penicillinase production continues and thus the antibacterial capacity of AMX is gradually decreased [65]. Even so, the antibacterial activity of HA-BDDE-AMX hydrogel against *S. aureus* was proven for at least the first high-risk 24 h, with log10 reductions (R) of 3.08 (6 h) and 0.38 (24 h).

### 2.7. Anti-Inflammatory Activity

**Figure 7:**
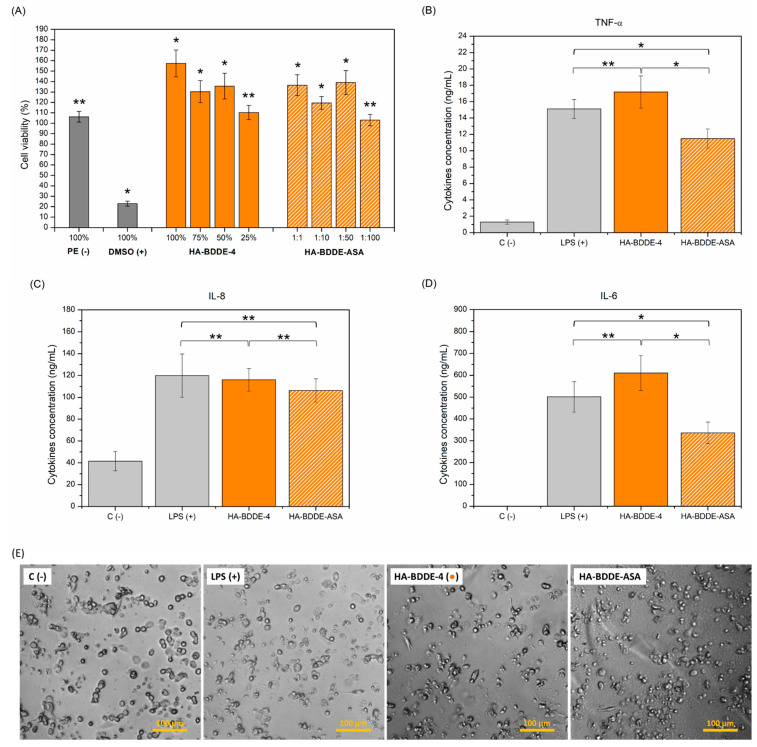
(**A**) THP-1 cell viabilities and (**B**) TNF-α, (**C**) IL-8, and (**D**) IL-6 cytokine levels for controls, HA-BDDE-4, and HA-BDDE-ASA. (**E**) Images of the morphology of the THP-1 cells obtained in the anti-inflammatory assay. * Significant differences (*p* < 0.05), ** non-significant differences (*p* > 0.05).

Inflammatory responses are natural reactions that typically arise when biomaterials are injected into the human body. In fact, inflammation is a beneficial process that helps to modulate damage and injuries and is governed by the regulation of pro-and anti-inflammatory cytokines [66]. The latter proteins play a key role in reducing inflammation, whereas great amounts of pro-inflammatory cytokines are responsible for excessive local chronic inflammation that endangers the therapeutic efficacy of implanted injectable hydrogels [67]. Interleukin-6 and -8 (IL-6, IL-8), and tumor necrosis factor alpha (TNF-α) are examples of pro-inflammatory cytokines [68]. Therefore, it is imperative to carefully control the levels of TNF-α, IL-8, and IL-6 and, thereby, relieve uncontrolled painful inflammation in patients. For this purpose, the anti-inflammatory properties of HA-BDDE hydrogels loaded with ASA (HA-BDDE-ASA) were evaluated (Figure 7) at 24 h, which is considered an adequate test time during which inflammation starts to become predominant in healing processes [69].

Before measuring anti-inflammatory activity, a cytotoxicity test was carried out with the same cell line as that used in the anti-inflammatory assay, since the false inflammatory responses that cells can produce if they die could have ruined our anti-inflammatory assay. In any case, the results shown in Figure 7A evidenced the cytocompatibility of HA-BDDE-4 and HA-BDDE-ASA (cell viabilities > 70%). Therefore, their highest concentration and dilution (100% and 1:1) were selected to determine their anti-inflammatory activity (Figure 7B–E), since neither the hydrogel itself nor the delivery of 1.35 mg of AAS per gram of hydrogel at 24 h seem to produce any toxicity. The HA-BDDE hydrogel did not show any anti-inflammatory activity considering the quantification of TNF-α (17.17 ± 1.96 ng·mL^−1^), IL-6 (610.33 ± 80.18 ng·mL^−1^), and IL-8 (116.04 ± 10.51 ng·mL^−1^). Nevertheless, a significant reduction (*p* < 0.05) in the cytokine levels of TNF-α (11.49 ± 1.16 ng·mL^−1^) and IL-6 (336.18 ± 49.30 ng·mL^−1^) compared to the LPS positive control (15.11 ± 1.16 ng TNF-α ·mL^−1^ and 501.31 ± 69.63 ng IL-6·mL^−1^) confirmed the anti-inflammatory response of HA-BDDE-ASA hydrogel, in spite of the non-significant decrease (*p* > 0.05) in the quantity of IL-8 cytokine (106.31 ± 10.65 ng·mL^−1^).

## 3. Conclusions

In this work, different HA-BDDE hydrogel formulations were successfully developed with different MoDs; we varied the synthesis parameters (reaction time and BDDE quantity) in order to obtain gels with desirable physicochemical properties. Considering the rheological, swelling, and stability results, hydrogels synthesized for 3 h (HA-BDDE-4, HA-BDDE-5, and HA-BDDE-6) were demonstrated to be the most promising for long-term application as injectable biomaterials. In addition, an exhaustive biocompatibility test based on the Ph. Eur. and UNE-EN-ISO international standards ensured the biological safety of the prepared hydrogels.

Moreover, HA-BDDE injectable hydrogels were validated as effective drug delivery platforms by the loading and subsequent sustained release of CFX, TCN, AMX, and ASA. Finally, drug-loaded hydrogels were demonstrated to have remarkable antibacterial and anti-inflammatory activities by their significant (*p* < 0.05) reduction in both harmful *S. aureus* bacterial infection and the levels of painful TNF-α, IL-6, and IL-8 pro-inflammatory cytokines. As a general conclusion, HA-BDDE injectable hydrogels are promising candidates for potential applications as sustained drug delivery systems for combatting the habitual adverse effects that jeopardize the applications of these medical devices.

## 4. Materials and Methods

### 4.1. Materials

HA-BDDE gels were synthesized using hyaluronic acid (HA, Contipro, Dolní Dobrouč, Czech Republic), 1,4-butanediol diglycidyl ether (BDDE, 95%, Sigma-Aldrich, Lyon, France) and sodium hydroxide (NaOH, 98%, Fisher Chemicals, Warsaw, Poland). Phosphate-buffered saline (PBS) at pH 7.4 was prepared with di-sodium hydrogen orthophosphate dodecahydrate (Na_2_HPO_4_·12H_2_O, 99%, Fisher Chemicals, London, UK), sodium dihydrogen orthophosphate dihydrate (NaH_2_PO_4_·2H_2_O, 99%, Fisher Chemicals, China), and sodium chloride (NaCl, 99%, Fisher Chemicals, Roskilde, Denmark). Hydrogels were purified and sustained drug release studies were performed with Iberlabo (Madrid, Spain) dialysis membranes (3500 Da and 12–14 kDa). Drug delivery studies of cefuroxime sodium salt (CFX, Sigma-Aldrich, Lyon, France), tetracycline hydrochloride (TCN, Sigma-Aldrich, Lyon, France), amoxicillin (AMX, 96%, Sigma-Aldrich, Lyon, France), and acetylsalicylic acid (ASA, 99%, Acros Organics, Lyon, France) were carried out. Regarding chromatographic assays, HPLC-grade acetonitrile (Chem-Lab, Barcelona, Spain), HPLC-quality water (LabKem, Colombo, Sri Lanka), and ammonium formate (Sigma-Aldrich, Lyon, France, 99%) were utilized. Deuterium oxide (D_2_O, 99.8 atom% D, Across Organics, Switzerland) was used for proton nuclear magnetic resonance (H^1^-NMR) spectroscopy analyses.

### 4.2. Synthesis of HA-BDDE Hydrogels

Hyaluronic acid was dissolved in a basic solution containing BDDE crosslinker at concentrations of 0.5 C, C, and 2 C. After vigorous stirring, the homogeneous mixture was reacted for 2 or 3 h to obtain HA-BDDE hydrogels (Table 2). The aqueous washing of hydrogels was performed by dialysis to remove unreacted crosslinker.

### 4.3. Physicochemical Characterization

#### 4.3.1. Proton Nuclear Magnetic Resonance Spectroscopy (^1^H-NMR)

The crosslinking of HA-BDDE hydrogels was investigated using the Bruker Advance NMR spectrometer (500 MHz, 25 °C). ^1^H-NMR spectra were obtained using deuterated water (33 mg/mL). Integrations of the signals at 1.60 and 1.90 ppm (*I^δH1.60^* and *I^δH1.90^*) were employed to measure the degree of modification (MoD) of HA-BDDE gels (Equation (1)).

(1) $$Degree\;of\;Modification\;\left(MoD\right)=\left(I^{\delta H1.60}/4\right)/\left(I^{\delta H1.90}/3\right)\times 100$$

#### 4.3.2. Fourier Transform Infrared Spectroscopy (FTIR)

A FTIR spectrometer (Nicolet Nexus, Thermo Scientific, Loughborough, UK) was employed to determine the infrared spectra of pure HA, BDDE, and synthesized HA-BDDE hydrogels. Spectra were recorded using KBr pellets in the 400–4000 cm^−1^ range at a 4 cm^−1^ resolution and 32 scans/spectrum.

#### 4.3.3. Rheological Properties

Rheological characterization was performed using an AR550 rheometer (TA instruments, New Castle, DE, USA) with a 40 mm diameter steel cone-plate. First, the viscosity of hydrogels with 0.01 to 1000 s^−1^ shear rate variations was obtained; then, viscosity was quantified through flux time sweeps for 300 s at 1 s^−1^. Finally, after fixing the strain at 1% in strain sweep tests (Figure S8), the elastic (G′) and viscous (G′′) moduli were measured with 0.01 to 10.00 Hz frequency variations.

#### 4.3.4. Low Vacuum Scanning Electron Microscopy (LVSEM)

After lyophilizing hydrogels (FreeZone 4.5 Liter Benchtop, Labconco, Kansas City, MO, USA) for 48 h at −50 °C under 0.1 mbar, the morphological characterization of hydrogels was realized. Images were acquired with the Quanta 250 FEG microscope (FEI, Eindhoven, The Netherlands) equipped with a large-field electron detector (LFD) at a 218× magnification, 12.2 working distance, 20 kV, 4.5 spot size, 18 °C, 100 Pa, and without humidity.

#### 4.3.5. Equilibrium Swelling Ratio

Known weights of lyophilized HA-BDDE hydrogels (*W_d_*) were immersed in simulated human physiological conditions (pH 7.4 PBS, 37 °C) for 48 h to guarantee that they reached an equilibrium swelling state. Then, the excess water was removed from the swollen hydrogels with a filter paper. Finally, the equilibrium swelling ratio of the hydrogels was measured according to Equation (2) by weighing the hydrogels over the time (*W_s_*) with a Pioneer analytical balance (10^−4^ sensitivity).

(2) $$Equilibrium\;Swelling\;Ratio=\frac{W_{s}-W_{d}}{W_{d}}$$

#### 4.3.6. In Vitro Degradation

Before performing the degradation test, HA-BDDE gels were weighted (*W_0_*). After that, the hydrogels were immersed in PBS simulating human physiological conditions (pH 7.4, 37 °C), and, at certain times (*W_t_*), their degradation was recorded with respect to the weight loss of W_0_. The remaining hydrogel mass was calculated using Equation (3).

(3) $$Remaining\;Mass\;\left(\%\right)=\frac{W_{t}}{W_{0}}\times 100$$

#### 4.3.7. Thermal Characterizations

A Mettler Toledo TGA/SDTA 851^e^ thermogravimetric analyzer (Giessen, Germany) was employed to perform thermal stability investigations of the synthesized gels in a 25–800 °C temperature range. Glass transition temperatures (T_g_) were determined using a Mettler Toledo DSC 822^e^ Differential Scanning Calorimeter (Giessen, Germany) in a 25–180 °C temperature range. Both tests were carried out under a 50 mL·min^−1^ nitrogen flux and 10 °C·min^−1^ heating rate.

### 4.4. Biocompatibility Test

#### 4.4.1. pH and Osmolality

The pH values of the synthesized gels were obtained using a SensION pH meter (HACH, pH31). Moreover, after removing air bubbles from the synthesized hydrogels, their osmolality was calculated using an Osmomat 030-D cryoscopic osmometer (Gonotec). Distilled water and 300 mOsmol·kg^−1^ of NaCl/H_2_O standard solution were used as blank and calibration solutions, respectively.

#### 4.4.2. In Vitro Cytotoxicity

The UNE-EN-ISO 10993-5:2009 international standard was used to evaluate the in vitro cytocompatibility of HA-BDDE gels via the extraction method. After incubating 1 × 10^−4^ CCL-171 cells for 24 h (5% CO_2_, 37 °C), extracts were added and then incubated again for another 24 h under the same seeding conditions. Lastly, a WST-1 colorimetric assay (Equation (4), 450 nm) was utilized to calculate the cell viability via the measurement of the absorbance of the test samples (*A*_450*m*_) and blanks (*A*_450*b*_). A total of 8 replicates per sample were tested.

(4) $$Cell\;viability\;\left(\%\right)=\frac{A_{450m}}{A_{450b}}\times 100$$

CCL-171 (ATCC), polyurethane film with 0.1% zinc diethyldithiocarbamate (ZDEC), high-density polyethylene (HDPE), and EMEM culture medium (supplemented with 10% SBF) were used as the cell line, control, extractor vehicle, and blank, respectively. HA-BDDE extract dilutions of 100%, 75%, 50%, and 25% were tested.

#### 4.4.3. In Vivo Acute Systemic Toxicity

Animal acute systemic toxicity tests were performed based on the UNE-EN-ISO 10993-11:2017 international standard. The response to the systemic injection of HA-BDDE hydrogels was evaluated in 10 male mice (Charles River, Calco LC, Italy); these were randomly divided equally into 5 negative control mice (cottonseed oil, Sigma-Aldrich, Lyon, France) and 5 treatment group mice (HA-BDDE hydrogel). In all cases, the general state and weight of each mouse, toxicity appearance, mortality, and clinical symptoms were recorded at 4 h, 24 h, 48 h, and 72 h after intraperitoneal injection (administration volume of 50 mL·kg^−1^).

### 4.5. Loading and Sustained Release of Drugs

Firstly, the degradation profiles of CFX, TCN, AMX, and ASA were determined in order to evaluate their stability in PBS. Then, drug loading on HA-BDDE hydrogels was performed (1.80 mg drug/hydrogel gram), with them being incorporated by absorption after the immersion of lyophilized hydrogels in drug-containing PBS for 3 days. Afterwards, drug-loaded hydrogels were dipped in simulated physiological medium (PBS, 37 °C) to quantify their cumulative release profiles by high-performance liquid chromatography (HPLC).

The HPLC system (Agilent 1260 Infinity II) was equipped with a DAD detector and a Zorbax Eclipse XCB-C18 column (5 μm particle size, 4.6 × 150 mm, 25 °C, 1 mL·min^−1^ flow rate). Samples were prepared by 1/10 dilution in PBS. A total of 10 mM of phosphate-buffered solution (eluent A) and acetonitrile (eluent B) at pH 3.6 were chosen as the mobile phases. Phases of 0–2 min, 10% B; 2–10 min 10–80% B; 10–11 min, 80% B; 11–13 min, 80–10% B; 13–15 min, 10% B gradient were used for the chromatographic separation of the analytes. Quantitative measurements at 273 nm were realized using the following calibration curves: CFX (Signal (mAU) = 38.277 C (mg/mL) + 1.752, R^2^ = 0.996), TCN (Signal (mAU) = 8.801 C − 2.793 (mg/mL), R^2^ = 0.991), AMX (Signal (mAU) = 2.521 C (mg/mL) + 4.333, R^2^ = 0.993), and ASA (Signal (mAU) = 4.198 C (mg/mL) + 2.825, R^2^ = 0.982). The AMX, CFX, ASA. and TCN retention times were 1.89, 6.01, 6.28, and 6.39 min, respectively.

The release mechanisms and kinetics of drugs from gels were determined using the Korsmeyer–Peppas mathematical model. In fact, the Korsmeyer–Peppas semi-empirical equation was the most suitable for the use of polymeric systems as hydrogels (Equation (5)).

(5) $$\frac{M_{t}}{M_{\infty }}=K\times t^{n}$$

*M_t_* and $M_{\infty }$ are the amount of drugs released at *t* time and in the equilibrium state; *K* and *n* are the release rate constant and diffusion coefficient. In the cylindrical tablets model, when *n* < 0.45 (Fickian), the drug release is governed by the diffusion mechanism.

### 4.6. In Vitro Antibacterial Activity

The antibacterial activity of non-loaded and AMX-loaded HA-BDDE hydrogels was assessed against that of *Staphylococcus aureus* (ATCC 6538). Specifically, each hydrogel (1 mL) was incubated with 1 × 10^5^–1 × 10^6^ Colony Forming Units (CFU) for 6–24 h at 37 °C; then, the amount of viable *S. aureus* bacteria was quantified. Finally, bacterial death (as reduction%) and antimicrobial activity (as *R* or *log*10 reduction) were calculated using Equations (6) and (7).

(6) $$Bacterial\;reduction\;\left(\%\;death\right)=\frac{B-A}{B}\times 100\%$$

(7) log10 reduction (R)=log10 (B)−log10 (A)

where *A* and *B* are the average viable CFU for the control and hydrogels after 6 h or 24 h of contact time. While the antibacterial activity of non-loaded HA-BDDE hydrogel was evaluated against that of the control, drug-containing HA-BDDE gel was analyzed against non-loaded hydrogel in order to determine the effects of drugs on *S. aureus*.

### 4.7. In Vitro Anti-Inflammatory Activity

Firstly, a CCK-8 cytocompatibility assay was carried out according to the UNE-EN-ISO 10993-5:2009 international standard in order to choose the most appropriate hydrogel concentrations for use in the anti-inflammatory test. Extracts at levels of 100%, 75%, 50%, and 25% were prepared for the HA-BDDE sample and 1:1, 1:10, 1:50, and 1:100 for the ASA-loaded HA-BDDE with RPMI-16 culture medium supplemented with 10% SBF extractor vehicle. Human monocytic leukemia (THP-1, ATCC), 10% dimethyl sulfoxide (DMSO), and high-density polyethylene (HDPE) were selected as the cell lone, positive, and positive controls. Later, 5 × 10^4^ THP-1 cells/well were induced from monocytes to macrophages with PMA (30 ng·mL^−1^) and seeded for 24 h (5% CO_2_, 37 °C). Subsequently, hydrogel formulations were added and incubated again for 24 h (5% CO_2_, 37 °C). After that period, 10 µL of CCK-8 reagent was used for the quantification of cell viability (n = 5, 450 nm).

After the cytotoxicity test, the highest cytocompatible hydrogel concentration was chosen to carry out the anti-inflammatory test using the THP-1 cell line. The same experimental procedure as that explained above was used, as well as the activation of monocytes using lipopolysaccharide (LPS, 100 ng·mL^−1^). The anti-inflammatory activity was evaluated by quantifying the levels of the interleukin-6 (IL-6), intereukin-8 (IL-8), and tumor necrosis factor alpha (TNF-α) pro-inflammatory cytokines by ELISA (Prepotech).

### 4.8. Statistical Analysis

If not specified, quantitative means ± standard deviations constitute a minimum of 3 replicates per group. One-way ANOVA (analysis of variance, 0.05 significance level with the Tukey test) was used to perform multiple comparisons between groups.

## Figures and Tables

**Table 2:** Summary of synthesized HA-BDDE samples and their crosslinking conditions.

Hydrogels	Reaction Time (h)	BDDE Concentration
HA-BDDE-1 (●)	2	0.5 C
HA-BDDE-2 (●)	2	1 C
HA-BDDE-3 (●)	2	2 C
HA-BDDE-4 (●)	3	0.5 C
HA-BDDE-5 (●)	3	1 C
HA-BDDE-6 (●)	3	2 C

## Data Availability

Not applicable.

## Supplementary Materials

The following supporting information can be downloaded at https://www.mdpi.com/article/10.3390/gels8040223/s1: Figure S1: Proton nuclear magnetic resonance (^1^H-NMR) spectra of HA-BDDE-1 hydrogel for MoD determination; Figure S2: Proton nuclear magnetic resonance (^1^H-NMR) spectra of HA-BDDE-2 hydrogel for MoD determination; Figure S3: Proton nuclear magnetic resonance (^1^H-NMR) spectra of HA-BDDE-3 hydrogel for MoD determination; Figure S4: Proton nuclear magnetic resonance (^1^H-NMR) spectra of HA-BDDE-4 hydrogel for MoD determination; Figure S5: Proton nuclear magnetic resonance (^1^H-NMR) spectra of HA-BDDE-5 hydrogel for MoD determination; Figure S6: Proton nuclear magnetic resonance (^1^H-NMR) spectra of HA-BDDE-6 hydrogel for MoD determination; Figure S7: FTIR spectra of BDDE, HA and HA-BDDE hydrogels between 4000-500 cm^−1^; Table S1: Acute systemic toxicity results of HA-BDDE-4 hydrogel and control group; Table S2: Physicochemical properties of CFX, TCN, AMX, and ASS drugs; Figure S8: G′ and viscous G′′ versus strain of HA-BDDE-1 for LVR determination by amplitude sweep.
