# Supplementary material for: Drug Delivery from Hyaluronic Acid–BDDE Injectable Hydrogels for Antibacterial and Anti-Inflammatory Applications
Source: Gels. 2022 Apr 6;8(4):223. doi: 10.3390/gels8040223 (PMC9033012; doi:10.3390/gels8040223)
Supplement: Supplementary file 1 [file gels-08-00223-s001.zip › gels-1647068-supplementary.pdf]

Drug Delivery from Hyaluronic Acid–BDDE Injectable Hydrogels for Antibacterial and Anti-Inflammatory Applications

Jon Andrade del Olmo [1,2], Leyre Pérez-Álvarez [2,3], Virginia Sáez Martínez [1], Sandra Benito Cid [1], Raúl Pérez González [1], José Luis Vilas-Vilela [2,3] and José María Alonso [1,*]

[1] i+Med S. Coop. Parque Tecnológico de Álava, Albert Einstein 15, nave 15, 01510 Vitoria-Gasteiz, Spain; jandrade@imasmed.comemail (J.A.O.); vsaez@imasmed.com (V.S.M.); sbenito@imasmed.com (S.B.C.); rperez@imasmed.com (R.P.G.)

[2] Grupo de Química Macromolecular (LABQUIMAC), Departamento de Química Física, Facultad de Ciencia y Tecnología, Universidad del País Vasco UPV/EHU, 48940 Leioa, Spain; leyre.perez@ehu.eus (L.P.-Á.); joseluis.vilas@ehu.eus (J.L.V.-V.)

[3] BCMaterials, Basque Center for Materials, Applications and Nanostructures, UPV/EHU Science Park, 48940 Leioa, Spain

* Correspondence: jalonso@imasmed.com

Supplementary material

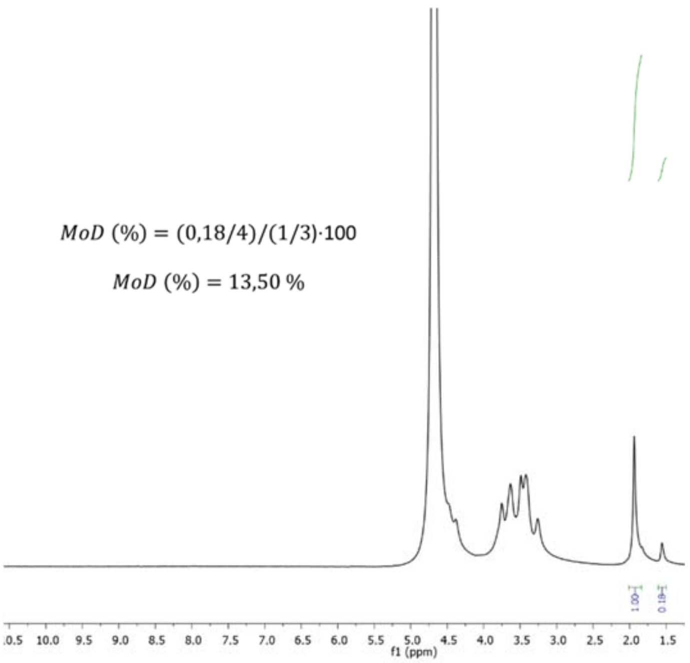

Figure S1. Proton nuclear magnetic resonance (^{1}H-NMR) spectra of HA-BDDE-1 hydrogel for MoD determination.

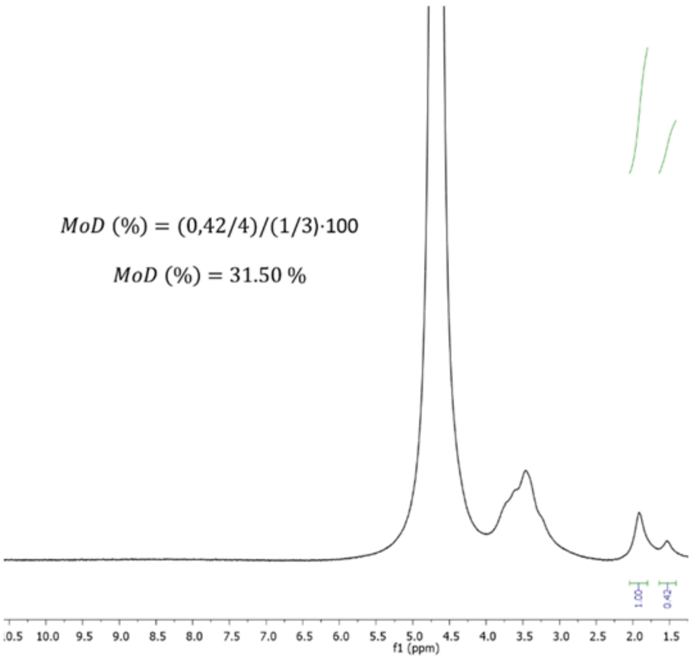

Figure S2. Proton nuclear magnetic resonance (^{1}H-NMR) spectra of HA-BDDE-2 hydrogel for MoD determination.

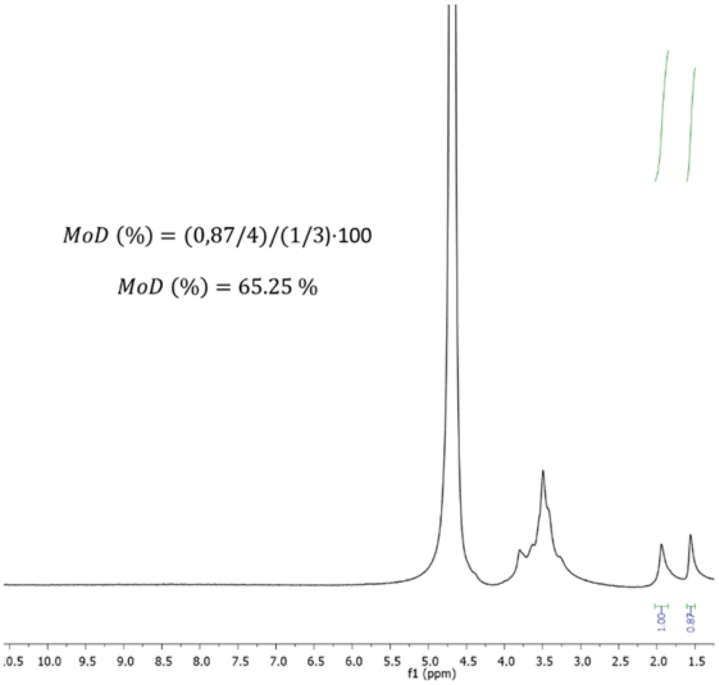

Figure S3. Proton nuclear magnetic resonance (^{1}H-NMR) spectra of HA-BDDE-3 hydrogel for MoD determination.

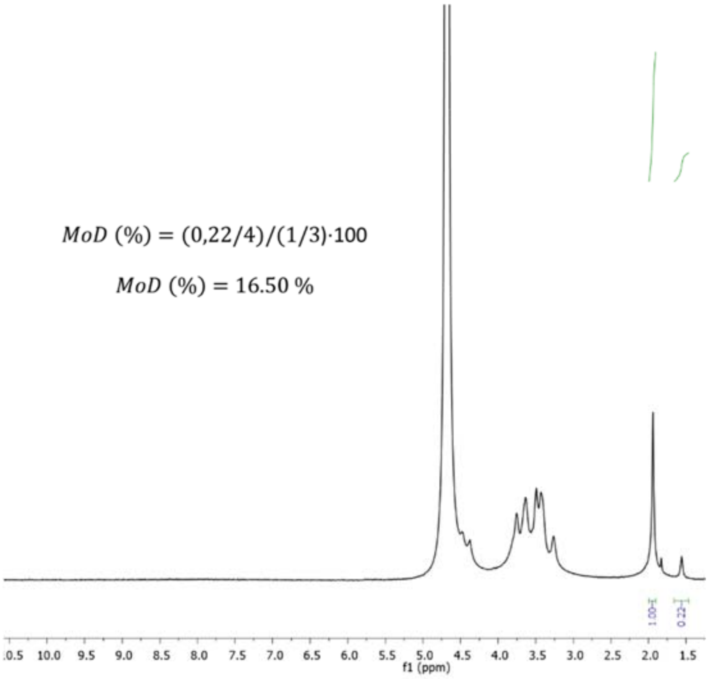

Figure S4. Proton nuclear magnetic resonance (^{1}H-NMR) spectra of HA-BDDE-4 hydrogel for MoD determination.

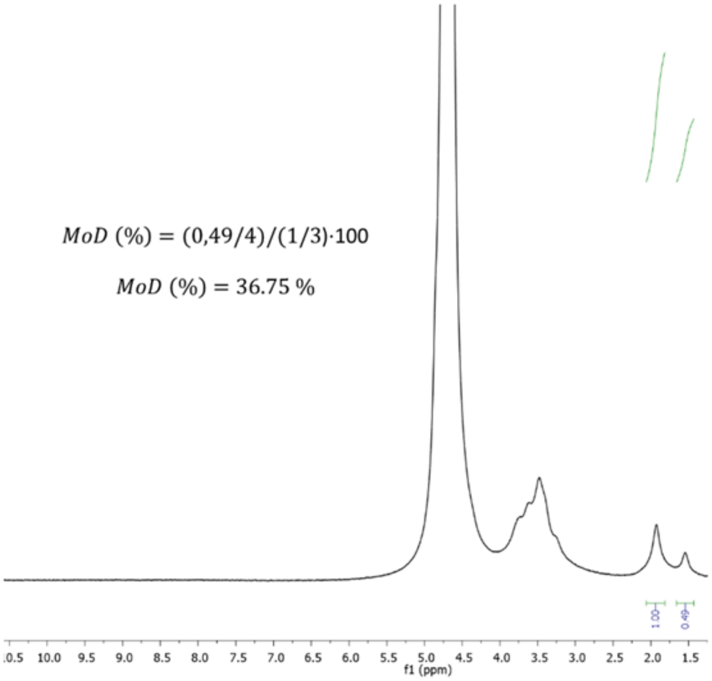

Figure S5. Proton nuclear magnetic resonance (^{1}H-NMR) spectra of HA-BDDE-5 hydrogel for MoD determination.

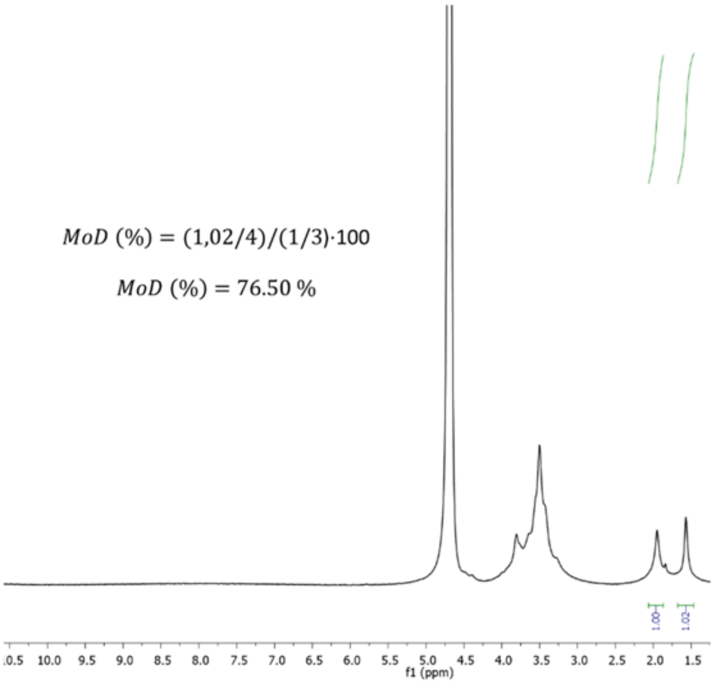

Figure S6. Proton nuclear magnetic resonance (^{1}H-NMR) spectra of HA-BDDE-6 hydrogel for MoD determination.

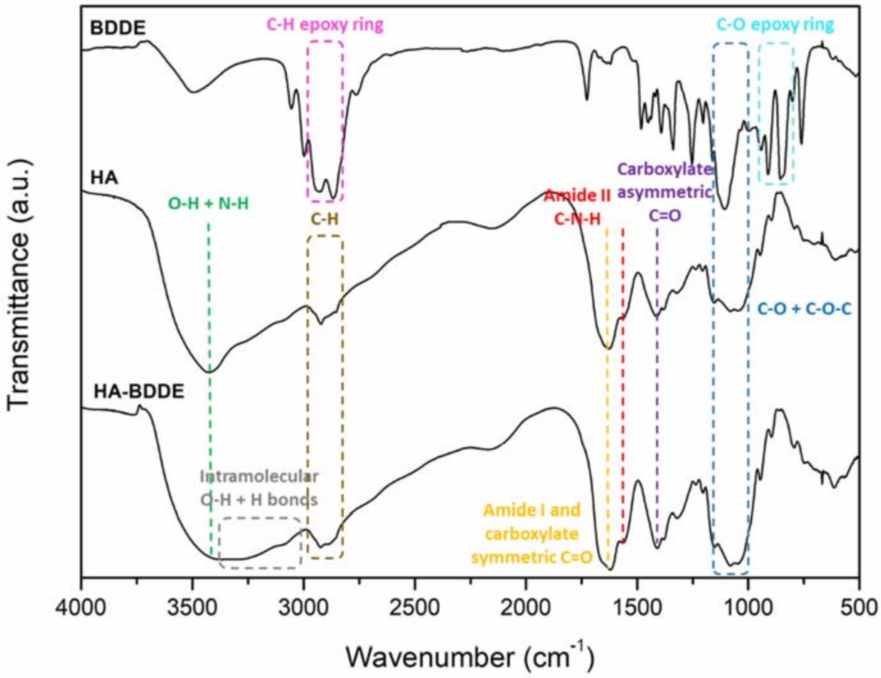

Figure S7. FTIR spectra of BDDE, HA and HA-BDDE hydrogels between 4000–500 cm^{-1}.

Table S1. Acute systemic toxicity results of HA-BDDE-4 (●) hydrogel and control group.

Animal number	Start weight (g)	End weight (g)	Clinical symptoms at different times (h)				
**HA-BDDE-4 hydrogel – Treated group**							
			**0**	**4**	**24**	**48**	**72**
1	23.6	27.5	0	0	0	0	0
2	22.8	27.4	0	0	0	0	0
3	20.4	23.2	0	0	0	0	0
4	21.5	25.3	0	0	0	0	0
5	23.4	27.0	0	0	0	0	0
**Cottonseed oil - Control group**							
**Animal number**	**Start weight (g)**	**End weight (g)**	**Clinical symptoms at different times (h)**				
			**0**	**4**	**24**	**48**	**72**
1	23.6	27.6	0	0	0	0	0
2	21.7	25.5	0	0	0	0	0
3	21.3	26.3	0	0	0	0	0
4	23.5	26.1	0	0	0	0	0
5	20.2	24.4	0	0	0	0	0

0: no symptoms.

Table S2. Physicochemical properties of CFX, TCN, AMX and ASS drugs.

Drug	Molecular weight (g/mol)	Water solubility (mg/mL, 25 °C)	log Kow	pKa	Net charge (pH 7.4)
CFX	446.4	> 100	− 0.80	pKa (1) 2.5	- -
TCN	480.9	22.0	− 1.30	pKa (1) 3.3<br>pKa (2) 7.7<br>pKa (3) 9.5	+
AMX	365.4	4.0	0.87	pKa (1) 2.4<br>pKa (2) 7.4<br>pKa (3) 10.6	-
AAS	180.2	3.3	1.19	pKa (1) 3.5	- -

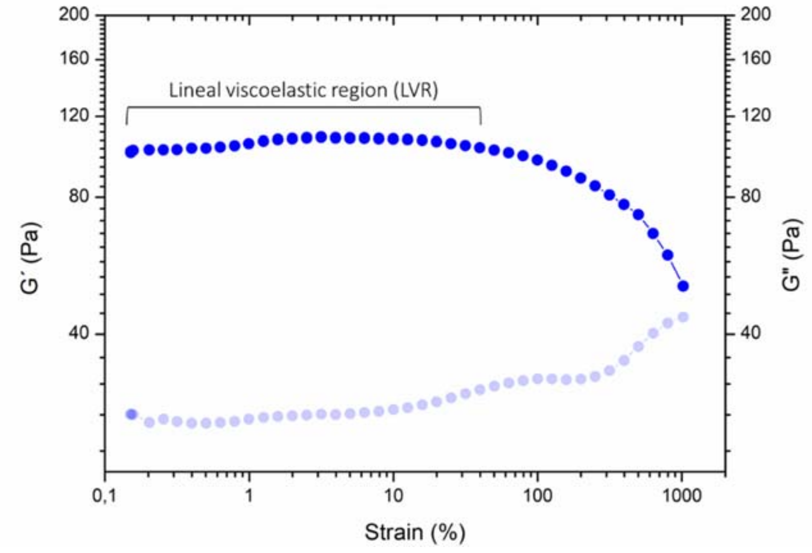

Figure S8. G´ and viscous G″ versus strain of HA-BDDE-1 (●) for LVR determination by amplitude sweep.
